# Competing Visual Cues Revealed by Electroencephalography: Sensitivity to Motion Speed and Direction

**DOI:** 10.3390/brainsci14020160

**Published:** 2024-02-04

**Authors:** Rassam Rassam, Qi Chen, Yan Gai

**Affiliations:** Biomedical Engineering, School of Science and Engineering, Saint Louis University, St. Louis, MO 63103, USA; rassam.rmg@outlook.com (R.R.);

**Keywords:** motion, speed, direction, visual, EEG, BCI, machine learning

## Abstract

Motion speed and direction are two fundamental cues for the mammalian visual system. Neurons in various places of the neocortex show tuning properties in term of firing frequency to both speed and direction. The present study applied a 32-channel electroencephalograph (EEG) system to 13 human subjects while they were observing a single object moving with different speeds in various directions from the center of view to the periphery on a computer monitor. Depending on the experimental condition, the subjects were either required to fix their gaze at the center of the monitor while the object was moving or to track the movement with their gaze; eye-tracking glasses were used to ensure that they followed instructions. In each trial, motion speed and direction varied randomly and independently, forming two competing visual features. EEG signal classification was performed for each cue separately (e.g., 11 speed values or 11 directions), regardless of variations in the other cue. Under the eye-fixed condition, multiple subjects showed distinct preferences to motion direction over speed; however, two outliers showed superb sensitivity to speed. Under the eye-tracking condition, in which the EEG signals presumably contained ocular movement signals, all subjects showed predominantly better classification for motion direction. There was a trend that speed and direction were encoded by different electrode sites. Since EEG is a noninvasive and portable approach suitable for brain–computer interfaces (BCIs), this study provides insights on fundamental knowledge of the visual system as well as BCI applications based on visual stimulation.

## 1. Introduction 

Electroencephalography (EEG) provides a portable and noninvasive solution for brain–computer interfaces (BCIs). By examining brain signals and features from a human user that are self-initiated or evoked by external stimulation, the intention of the human can be inferred and used to control external devices. Typical visual BCI protocols are based on transient stimulation that evokes P300’s [1] or steady-state visual evoked potentials (SSVEPs) [2], both providing the human user with a limited number of choices. For example, various chessboard patterns may correspond to the commands of “left”, “right”, “go”, and “stop” for BCI wheelchair users [3]. By directing the central visual field to a particular pattern, distinct brain signals can be recorded to infer the human’s intention. Alternatively, visual objects with different directional preferences can be used to decode the user’s spatial attention [4]. Regardless of the stimulation protocol, the general mechanism of a SSVEP-based BCI remains the same: a distinct set of visual stimuli should evoke consistently distinguishable EEG features.

While existing BCI applications have made significant progress in utilizing visual stimuli for interpreting users’ intention, most of them focus on relatively simple and/or unidirectional stimuli. This is partly due to the fact that EEG features elicited by more complex and competing visual cues have not been adequately explored. Using BCI wheelchairs as an example, while decoding the user’s spatial attention has been successfully implemented as mentioned above, e.g., which direction the user would like the wheelchair to move, there has been no attempt to decode user intentions that are linked to movement speed, e.g., how fast the user would like the wheelchair to move. Although it is theoretically possible to use different visual patterns (such as the above-mentioned chess-board patterns) to represent various speeds, this type of stimulation protocol is unnatural. Ideally, users should be able to control both the direction and speed of a wheelchair by selectively fixing their gaze (i.e., their central visual field) at the preferred visual stimulus, among a set of stimuli, that has the desired direction and speed.

Therefore, in the present study, we examined the potential of constructing SSVEP-based BCIs using two important visual motion cues—speed and direction. We particularly focused on the comparison of EEG sensitivities to the two cues as elicited by each individual human, because different people may have different cue preferences. To create competing cues, the motion speed and direction of the same object were randomly and independently chosen. This design will, of course, make subsequent signal classification more difficult than fixing one cue each time and only varying the other. The latter approach may generate bias because two separate datasets are recorded at different times, each for a particular cue, while we examined the same dataset containing both cues for each subject and EEG electrode.

Because the visual object was moving on a computer monitor in front of the human subject, a natural variation would be whether we allow the human subject to track the object with their eyes. It is known that ocular signals can be present in EEG recordings, and there are various methods to record or remove the electrooculogram (EOG) [5]. From the perspective of examining brain signals associated with speed and direction cues, it is best to keep the eyeball fixed or have the EOG removed. On the other hand, in the real world, our eyes are rarely still, and BCI applications that prohibit users from tracking moving objects would seem unnatural. If including the EOG in EEG signals improves classification performance, there is no reason why we should not do so. Unfortunately, few studies have examined speed sensitivity when the eyes are smoothly pursuing a moving target [6].

In addition, machine learning classification algorithms typically follow signal acquisition and feature extraction to decode the user’s intention in BCI applications [7,8]. For example, given a piece of EEG recording, can we tell in which direction the user would like the wheelchair to move? In this sense, a classification algorithm will yield a specific outcome, such as left or right. Commonly used classification algorithms for ongoing EEG signals include linear discriminant analysis (LDA), support vector machine, k-nearest neighbor, template matching, artificial neural networks [3,9,10], etc. The present study chose the LDA algorithm because high-dimension signals (i.e., the entire temporal waveform over half seconds) are used as the input to the classifier. LDA is a commonly used dimension reduction method [11] and has been applied to classify event-related potentials by other researchers in the past [12,13,14].

In this study, we first recorded the EEG and examined its sensitivity to the two visual cues under a eye-fixed condition. We also repeated the entire experiment with each human subject while the subjects were required to track the moving object with their gaze. To ensure that the subjects followed the instructions, they wore eye-tracking glasses during both experimental conditions, and their gaze data were screened afterwards. In addition, we introduced a large number of “classes” in the task, i.e., 11 speeds and 11 directions, which is rare compared with previous EEG studies, such as those discriminating two [15] or three speeds [16] and six directions [4], etc. We will first present speed and angle classification results under the eye-fixed condition. We will then show the results obtained under the eye-tracking condition as a comparison.

## 2. Methods

### 2.1. General Setup

Thirteen subjects (age 19–25) were paid to participate in this study. Subjects had either normal or corrected-to-normal vision. For the latter, a screening procedure was performed to make sure that short-sightedness, if any, was mild enough to enable eye tracking (e.g., we had excluded a couple of subjects with more severe short-sightedness who had failed the eye-tracking screening.) The experimental protocol was approved by the Institutional Review Board of Saint Louis University (Protocol #29002).

**Figure 1:**
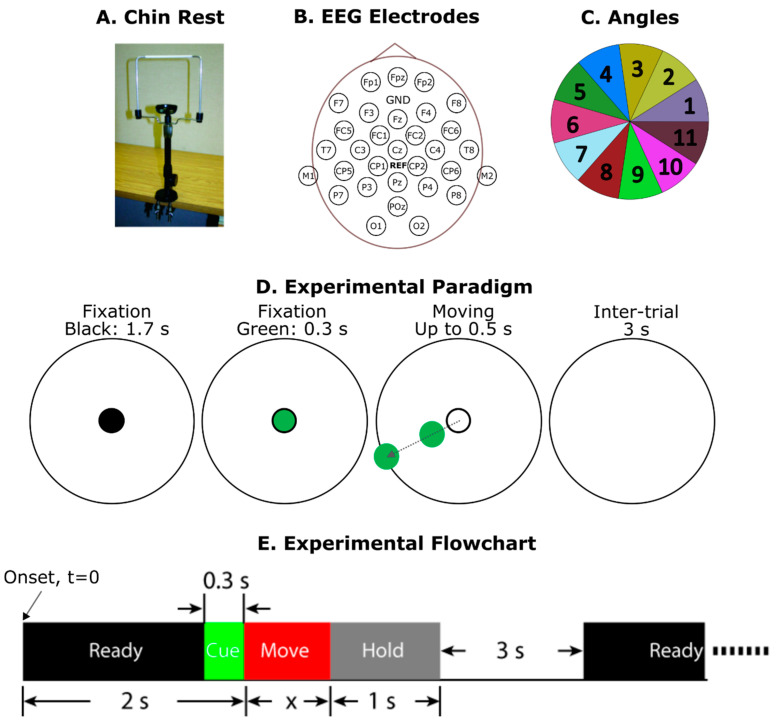
(**A**) Chin rest to support to subject’s head. (**B**) EEG electrode map. GND, ground electrode. REF, reference electrode CPz. (**C**) The 11 angles to be classified. In each trial, the angle of movement was randomly selected and was then put into one of the 11 directions to be classified. (**D**,**E**) Experimental paradigm (**D**) and flowchart (**E**), respectively. At the beginning of each trial, there was a 2 s fixation period. During the last 0.3 s of fixation, the black fixation point turned to green to signal that the motion would begin soon. In the “eye-fixed” condition, the subject was asked to look at the center, except during the intertrial intervals. In the “eye-tracking” condition, the subject was asked to track the moving green circle.

During the experiment, subjects were sitting in a chair with a head rest to support their chin (Figure 1A; SR Research, Kanata, ON, Canada, Eye Link), wearing eye-tracking glasses (Tobii, Stockholm, Sweden). A 27-inch desktop computer screen was placed in front of them on a desk. The distance between the subject and the screen was 50 cm. All subjects with normal or corrected vision were recorded under the same setting, ensuring uniformity in screen positioning, distance, and viewing angle. At the beginning of each experimental session, gaze calibrations were performed with the eye tracker, during which visual objects were presented on the four corners of the computer screen. Recorded gaze data allowed us to double check if the subject had followed instructions during different experimental conditions; gaze data were not used in any of the signal classification tasks.

Meanwhile, EEG signals were measured with a 32-channel portable system (Figure 1B; eego-sports; ANT Neuro, Hengelo, The Netherlands), including a head cap, an amplifier, and a Windows tablet computer. A StimTracker (Cedrus, San Pedro, CA, USA) was used to mark the stimulation onsets and label the EEG trials.

### 2.2. Experimental Paradigms

Figure 1D,E shows the trial-by-trial procedure. The larger circle on the screen (Figure 1D) had a diameter of 25 cm. Given that the subjects’ eyes were 50 cm away from the screen, the end angle of the eye motion was 14°. At the beginning of each trial, there was a 2 s fixation period. During the last 0.3 s of fixation, the black fixation circle turned to green to signal that the motion would begin soon. Motions could be in any random direction with a speed randomly selected from 11 speed values. During angle classifications, the space inside the large circle was partitioned into 11 directions to match the number of speeds for a fair comparison (Figure 1C).

The slowest speed on the computer screen was 25 cm/s, which corresponds to 28 °/s for the human gaze. Each speed interval was intended to be twice as fast as the previous speed; however, drawing the circle along the moving trace (Figure 1D) may have taken some extra time (eight drawings were performed for each motion). Therefore, we also recorded the actual beginning and end time for each actual motion, with the slowest speed taking about 0.5 s and the fastest speed taking 0.1 s.

After the completion of the motion, the object was held on the circle for 1 s (Figure 1E) before it disappeared. Each trial was followed by a 3 s intertrial interval, during which the subject was allowed to relax the eyes. It was critical to have the 1 s holding period so that the EEG response to the moving object would not be affected by eye motions during the intertrial interval.

**Figure 2:**
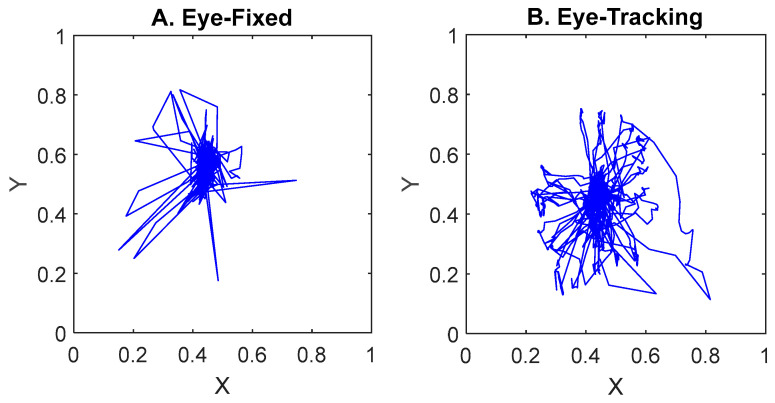
Examples of continuous eye traces monitored with Tobii eye-tracking glasses for Subject S1. (**A**) Gaze was fixed at the center of the image and was not allowed to move during each trial. (**B**) Gaze was tracking the motion of the object. The standard deviations of traces from center were 0.017 and 0.060 for (**A**) and (**B**), respectively. In both conditions, the subject was allowed to relax their eyes during intertrial intervals.

In the “eye-fixed” condition, the subject was required to look at the center without moving their gaze, except during the intertrial intervals. In the “eye-tracking” condition, the subject was required to track the moving green circle. Figure 2 shows examples of eye traces during the two conditions. For each subject, we obtained 15 sessions for the eye-fixed and 15 sessions for the eye-tracking condition. Each session contained 33 trials, with 3 trials for each of the 11 speeds and random directions. Therefore, each speed or direction contained 45 trials.

### 2.3. EEG Signal Processing and Classification

Figure 1B shows the EEG electrode map. The ground electrode was located in between Fpz and Fz. Electrode CPz was the reference electrode during the recordings, set as the default by the amplifier. A common-montage mode [17] was then applied in the signal-processing stage. Briefly, raw recordings from each electrode were subtracted by the average of all 31 electrodes. An independent component analysis (ICA) approach was applied to remove EEG artifacts [18], mostly eye-blinking signals. The sampling frequency of the amplifier was 500 Hz. A 50th-order FIR filter (0.1–13 Hz) was applied to the signal as preprocessing so that delta, theta, and alpha waves were included. We found that extending to higher frequencies did not improve the classification results.

**Figure 3:**
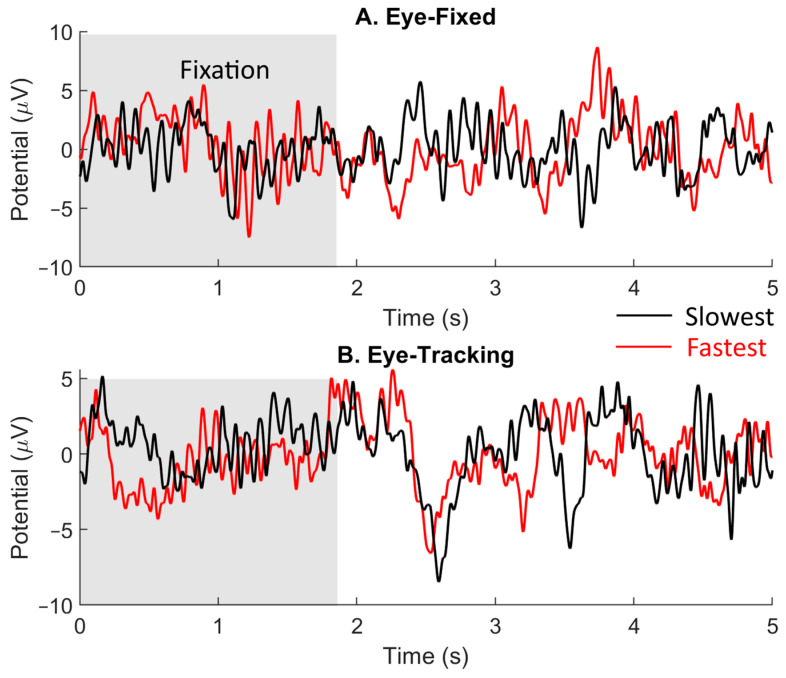
Example VEPs used for speed classification obtained from Subject S5, Electrode P4. The shaded area represents the 2 s fixation period. Each trace was an average over 45 trials for a fixed speed with any angle. Black traces were responses to the slowest speed (Speed #1) and red traces were responses to the fastest speeds (Speed #11). The slowest motion took about 0.5 s following t = 2 s.

First, we extracted the visual evoked potentials (VEPs) after the fixation onset. Figure 3 shows examples of VEP traces during the eye-fixed and eye-tracking conditions recorded using the same electrode (P4) for subject S5. Briefly, the preprocessed (including eye-blinking removals and bandpass filtering) EEG signals were extracted and averaged over 45 trials. The gray area between 0 and 2 s indicates the fixation period, during which the eyes were not allowed to move under either eye condition. Motions started at 2 s and the slowest one would finish at 2.5 s. In the eye-tracking condition, the eyeball could start tracing the object with a latency from less than 100 ms to more than 300 ms after the onset of the object’s motion [19]. The end of the eye tracking depended on the speed of the object and usually finished within 2 s in our study, depending on the subject.

Note that both motion speed and direction could vary randomly and independently. Next, we examined whether the motion speed or direction of the object could be decoded from the recorded VEP using machine learning and compared decoding performance across the eye-fixed and eye-tracking conditions. LDA was applied to the portion of VEP signals that occurred during the motion period (2–2.5 s, Figure 3). LDA aims to find a linear combination of features that separates two or more classes of objects or events. Considering that neural responses in the primary visual cortex (V1) have short response latencies that vary between 70 and 97.9 ms [20], our 500 ms response window should generally correspond to the motion of the visual object. Because the maximum signal dimension that can be optimized by the LDA algorithm is restricted by the number of samples (i.e., *n* = 495 when all motion trials were included) [21], the VEP was down-sampled to 25 samples/s to reduce the signal dimension.

Speed or direction classification accuracy at each electrode was then subsequently derived. To improve the signal-to-noise ratio, a leave-three-out cross-validation approach [22] was used to provide the training and test VEPs to the classifier. Briefly, each time, three trials obtained with the same class (i.e., speed) were randomly selected from the 45 trials and averaged to serve as a “test VEP”. Meanwhile, 100 training VEPs for each class were randomly formed from the pool, excluding trials that had been selected as the test VEP. After training the classifier, a prediction was made for the test VEP regarding the speed or direction of the object. The prediction could be correct or incorrect, which was the validation process. This procedure was repeated 100 times to obtain classification accuracy $\hat{\gamma }$.

### 2.4. Statistical Analysis

The majority of data created in this study were in the form of classification performance $\hat{\gamma }$, which was a percentage describing the correct classification rate. For this type of percentage measurements, a confidence interval (*CI*) approach is the standard in examining its statistical significance. Specifically, the significance of $\hat{\gamma }$ was used in computing the *CI* as

(1) $$CI=\pm z^{*}\sqrt{\frac{\hat{\gamma }(1-\hat{\gamma })}{n}}$$

with a significance level of *α* < 0.01 [23]. *n* is the number of trials, and z* is the standard normal. If $CI-\hat{\gamma }$ is greater than chance performance (e.g., 9.1% for 11 speeds or angles), the significant level is reached. When examining the significance of all 31 electrodes, a Holm–Bonferroni correction was applied to correct for multiple measurements.

When we compared the grand performance of all 31 electrodes across the two cues (speed vs. direction), a two-tailed *t*-test was applied to the two sets of correct detection rates to examine if one cue dominated the performance of the other.

## 3. Results

### 3.1. Gaze Monitoring

In this study, we compared the sensitivity of the EEG response to competing cues (i.e., speed vs. direction) of a moving object under eye-fixed and eye-tracking conditions. Eye-tracking glasses were used to make sure that the subjects generally followed the instructions, which was the case for the 13 subjects shown here. Results from one more subject have been excluded from the study since that subject sometimes involuntarily tracked the object during the eye-fixed condition.

Figure 2 shows examples of eye-tracking traces. During the eye-fixed condition, the traces were mostly confined to the center of the circle. Occasional motions away from the center were relaxations during intertrials, which was allowed. In comparison, the eye-tracking condition showed systematic gaze motions in various directions. The recorded end points usually formed an ellipse with the longitudinal axis being vertical, rather than a perfect circle, due to the design of the eye-tracking glasses.

To quantitatively separate the two conditions, we computed the standard deviations of eye traces from the center (geometrical mean of 2D traces). In the eye-fixed condition, traces typically had a standard deviation considerably below 0.030, whereas the tracking condition yielded much higher values, as the one in Figure 2B. Gaze data were only meant for monitoring purposes and were not used in any of the EEG classifications.

### 3.2. Visual Evoked Potentials

Figure 3 shows examples of VEPs for one subject obtained with the fastest (red) and lowest (black) speeds under the eye-fixed (A) and eye-tracking (B) conditions. Each trace was an average of 45 trials with the same speed. All conditions were the same during the first 2 s of fixation (gray area). The motion started at 2 s and the slowest speed took 0.5 s to finish, followed by a 1 s holding period. That is, the holding period started earlier for faster-motion-speed trials. Positive and negative evoked peaks were clearly seen under both eye conditions during the motion period. The portion of the VEP during 2.0–2.5 s was extracted and used for speed and/or direction classification.

For the eye-tracking condition, after the 1 s holding period, large VEPs occurred due to the eyeball moving back to the center (Figure 3B, 3.5–4 s). The evoked peaks occurred later for slow-speed trials because the holding period (1 s) ended later than in fast-speed trials. In addition, according to previous studies [19], the human gaze can move with a latency from tens to hundreds of milliseconds for near and far targets. Therefore, eyeball motions may have occurred during the 0.5 s used for the following VEP classification.

It should be pointed out that the VEPs in Figure 3 were constructed based on motion speed, regardless of direction. The VEPs may look different when being extracted according to motion direction using the same set of EEG recording waveforms.

### 3.3. Speed Classification, Eye-Fixed Condition

**Figure 4:**
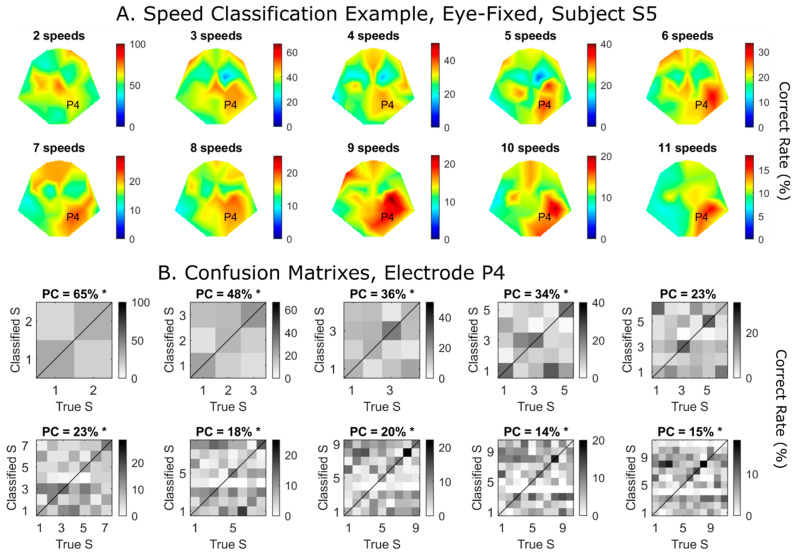
(**A**) Examples of classification performance topographies for motion speeds under “eye-fixed” condition for Subject S5, starting with the slowest speed. The “two speeds” plot shows classification performance of two slowest speeds. The rest are plots including successively faster speeds. Midpoint of color scale always corresponds to chance level with the number of classes specified. (**B**) Confusion matrixes for electrode P4 as an example. The *x*-axis is the true speed, with “1” being the slowest speed. The *y*-axis is the classified speed. Any decision falling on the diagonal is a correct decision. Asterisks represent statistical significance (*CI* test, *p* < 0.01). Note that in both (**A**,**B**), color scale is adjusted so that center of scale corresponds to chance performance, e.g., 50% for two speeds and 9% for eleven speeds. PC, percentage of correct decisions.

Figure 4A shows classification performance topography for a representative subject, S5, under the eye-fixed condition. Warm colors represent higher classification accuracies. The “two speeds” condition only includes VEP responses to the two slowest moving speeds. Here, chance performance is 50%. Similarly, “three speeds” includes the three slowest speeds, with chance performance being 33.3%. The last figure (“11 speeds”) is the one in which all recorded VEP responses are included. Here, the middle of each color bar (green) is set to be the chance value. Although the color darkens with the number of speeds included, absolute values are decreasing.

To explain how classification accuracy was computed, we chose one electrode (P4) and showed confusion matrixes for this electrode in the figure above (Figure 4B). Here, the columns are the true speed numbers, with “1” being the slowest and “11” being the fastest speeds, respectively. The rows are the classified speed indexes. Dark colors falling on the diagonal are correct classifications. The title of each small plot shows the percentage of correct values corresponding to the P4 electrode highlighted in Figure 4A. Again, the color bars are set to have chance performance in the middle of the color scale.

It can be seen that relative classification patterns (i.e., confusion matrixes) remained somewhat consistent as more speeds were added. For example, Speed #3 (i.e., the third slowest speed) had the highest classification rate when only the slowest three speeds were included (Figure 4B, upper row, second plot). Even with more speeds added, Speed #3 remained better than Speeds #2 and #1. Another example is that Speeds #6–9 showed relatively high performance throughout the last three plots. In other words, we could more or less predict the patterns for fewer speeds based on the confusion matrix for “all speeds” (Figure 4B, lower rightmost), although the exact value of each cell decreased with more speeds included.

For Electrode P4, we also computed its statistical significance using the *CI* approach (see Section 2.4). An asterisk in the title indicates significant performance (*p* < 0.01, Figure 4B). In this example, all but the six-speeds condition showed significant performance with P4. We should point out that speed classification was performed regardless of motion direction, which was randomly chosen in the 360° domain.

**Figure 5:**
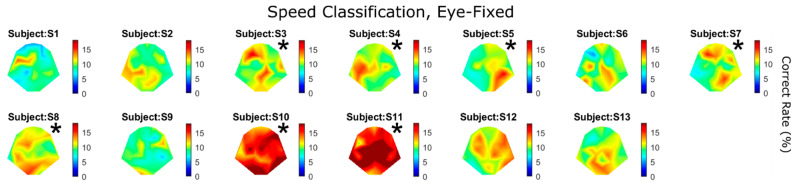
Speed classification results for all subjects under “eye-fixed” condition. There were a total of 11 speeds involved in the classification; therefore, chance performance was 9%. Asterisks indicate statistical significance (*CI* test, *p* < 0.01, with Holm–Bonferroni correction). The best electrode performance for all subjects was 16 ± 3.7%. Asterisks represent statistical significance (*CI* test, *p* < 0.01).

Figure 5 shows the classification performance for all 11 speeds and 13 subjects. For example, the very last heatmap plot in Figure 4A (11 speeds) is again shown here as “Subject S5”. Again, warm colors indicate higher classification performance. Asterisks indicate significant detection performance using the *CI* approach (*p* < 0.01; Equation (1)). Since there were multiple comparisons, a Holm–Bonferroni correction was applied during statistical tests. Most subjects had more than one electrode showing sensitivity to motion speed (seven out of thirteen subjects reached statistical significance), whereas Subjects S10 and S11 demonstrated strikingly high performance with a large number of electrodes. Later, we will show that those two subjects were the only ones whose sensitivity to motion speed was higher than their sensitivity to direction (Figure 8).

### 3.4. Direction Classification, Eye-Fixed Condition

**Figure 6:**
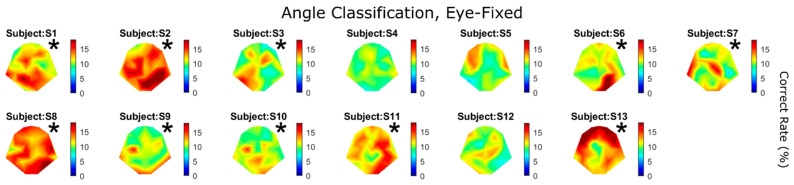
Direction classification results for all subjects under “eye-fixed” condition. There were a total of 11 angles involved in the classification; therefore, chance performance was 9%. The best electrode performance for all subjects was 17 ± 3.1%. Asterisks represent statistical significance (*CI* test, *p* < 0.01).

The results shown in Figure 4 and Figure 5 above were based on VEPs classified using motion speed. For the same speed, motion could occur in any random direction. Alternatively, VEPs can also be classified according to motion direction, regardless of speed; that is, according to how well we can tell in which direction the object moved based on a given piece of EEG. Figure 6 shows that 10 out of the 13 subjects had significant detection performance for motion direction. Interestingly, the two subjects who showed the highest sensitivity to motion speed (Figure 5), S10 and S11, were no longer the best two subjects for motion direction (Figure 6). On the other hand, multiple subjects who failed to show significant performance in the speed classification task (S1, S2, S6, S9, and S13; Figure 5) showed significant direction sensitivity (Figure 6).

### 3.5. Classification Results under the Eye-Tracking Condition

**Figure 7:**
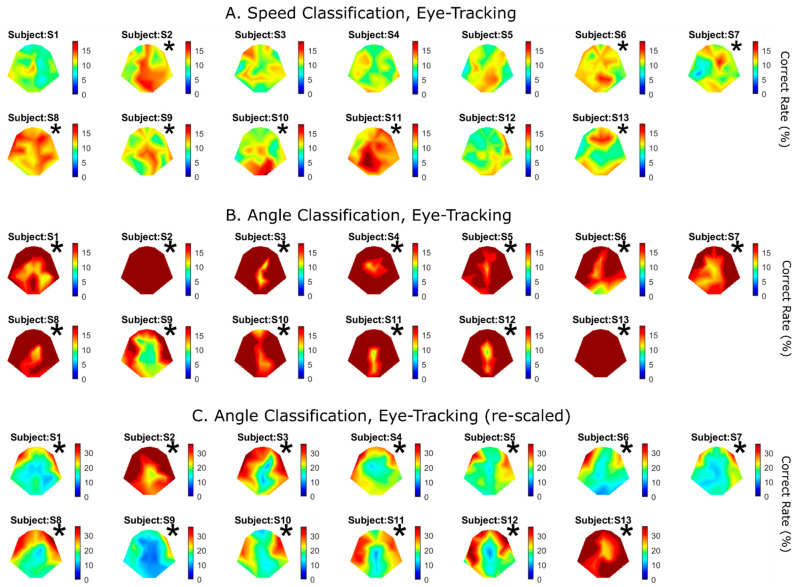
Speed (**A**) and angle (**B**) classification results under the eye-tracking condition. The format is the same as in Figure 5 and Figure 6. The best electrode performance for all subjects was 16 ± 1.8% for speed and 40 ± 8.5% for angle. (**C**) is the same as (**B**) except that the color scaled is doubled (maximum color is 36%) to expose the best electrodes. Asterisks represent statistical significance (*CI* test, *p* < 0.01).

As can be seen in Figure 3, moving the eyeballs can greatly affect VEP shape, presumably due to the presence of EOG. For the eye-tracking condition, we again limited VEPs to the duration of the motion (2–2.5 s). Figure 7A shows speed classification performance, which is to be compared with Figure 5 under the eye-fixed condition. Interestingly, allowing the eyeballs to freely move to track the object did not necessarily improve classification performance. Although nine out of thirteen subjects showed significant performance, the two subjects who showed superior performance under the eye-fixed condition (S10 and S11; Figure 5) exhibited lower sensitivity to speed under the eye-tracking condition (Figure 7A).

Classification performance was strikingly better for motion direction when the subjects were required to track the object (Figure 7B), in comparison with the eye-fixed condition (Figure 6). Here, the maximum color is capped at 18%, because chance performance was 9% for 11 angles. All 13 subjects showed highly significant performance, and maximum performance ranged from 28.0% to 62.5%. To expose the best-performing electrode sites, Figure 7B is replotted in C with a doubled color scale, i.e., maximum performance is capped at 36%. The best electrodes are mostly the frontal–lateral ones (Figure 7C).

**Figure 8:**
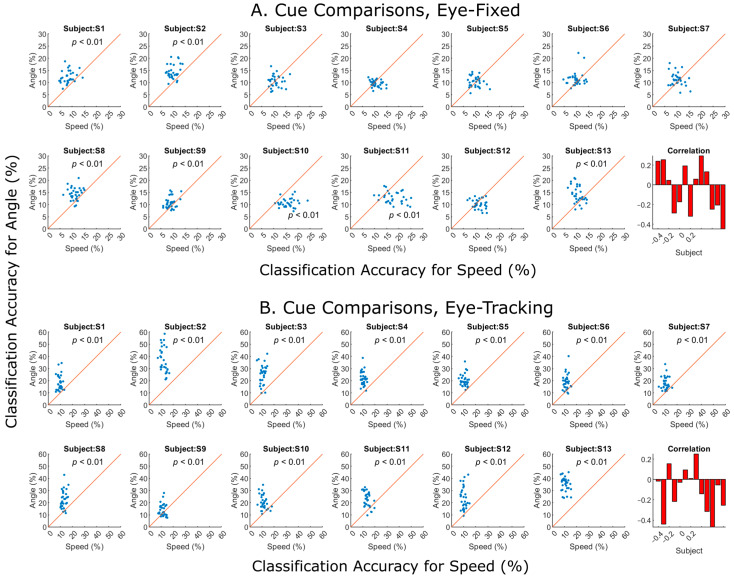
Speed vs. angle classification results under both conditions. Each dot represents the detection performance of 1 of the 31 electrodes. Under the eye-fixed condition (**A**), different subjects showed different preferences for the two cues. Subjects S1, S2, S8, S9, and S13 showed significantly better performance for direction (*t* test, *p* < 0.01), whereas Subjects S10 and S11 had better performance for speed (*t* test, *p* < 0.01). Under the eye-tracking condition (**B**), direction classification was predominately better for all subjects (*t* test, *p* < 0.01). The last panel in each figure with red bars shows the Pearson correlation coefficient between the two cues for each subject.

To directly compare EEG sensitivity between the two visual cues, motion speed and direction, a scatter plot was generated for each subject under both the eye-fixed (Figure 8A) and eye-tracking (Figure 8B) conditions. Each dot represents one electrode. A two-tailed *t*-test was applied to compare sensitivity to the two cues across all the electrodes for each human subject. Under the eye-fixed condition, only Subjects S10 and S11 showed significantly better performance for the speed cue (Figure 8A). The rest of the subjects either favored the direction cue or showed no preference. Needless to say, under the eye-tracking condition, all subjects showed significantly better performance for the direction cue (Figure 8B).

One may then ask the question: are speed and direction encoded by the same set of EEG electrodes or by different ones? For example, the dots in Figure 8A for Subject S7 seem to form a negative correlation, indicating that electrodes which performed better with one cue did worse with the other cue. Therefore, the last panel in each figure with red bars plots the Pearson correlation coefficient between the two cues for each subject. Overall, negative correlations seem to be more prevalent than positive correlations, especially under the eye-tracking condition (Figure 8B, lower rightmost panel). In addition, EEG data are characterized by various signal features, including electrode positioning and frequency components. The application of different signal processing methods can result in distinct outcomes.

## 4. Discussion

### 4.1. Neural Coding of Motion Speed and Direction

As stated by Pasternak and Tadin [24], “…the direction and speed of target motion are among the most important encoded stimulus properties, revealing many parallels between psychophysical and physiological responses to motion.” Neural mechanisms for coding motion direction have long been studied along the visual pathway in the mammalian brain. There are orientation columns in the primary visual (striate) cortex that encode the directions of movements in terms of direction-tuning curves [25,26]. Similar observations are also common in extrastriate cortical areas such as V3 [24].

The speed of a moving object is also a prime dimension studied in visual neuroscience. Speed perception is a critical survival skill for humans and other species to properly react to surrounding events. It has been suggested that speed can be perceived separately from distance and time [6]. However, there have been contradicting views on how speed is encoded, i.e., whether speed is a feature perceived independently from spatial and temporal features. One hypothesis is that speed is directly coded by neurons showing speed tuning [27]. Most studies have been conducted in monkeys and have shown single neurons with speed tuning that do not directly code temporal or spatial frequency [28,29,30,31,32]. Direct speed tuning in MT/MST has also been identified in humans using fMRI [33], at least for high-contrast stimuli. On the other hand, many studies suggest that speed is first encoded by neurons that detect spatial and temporal frequencies [34,35,36,37,38,39,40], and thus further processing is needed to extract true speed information.

In our study, because we used a single moving object (i.e., a small circle) without any spatial and temporal frequency features, the result indicated direct sensitivity to speed.

### 4.2. The Experimental Design vs. BCI Applications

The present study examined EEG sensitivity to two competing motion cues—speed and direction. The effect of eye movements on classification performance was examined while the subject was allowed or forbidden to track the moving object. Two aspects of the experimental design made classification especially challenging.

First, signal classification was performed for each cue while the other cue was varied randomly and independently. Regardless of the choice of machine learning algorithms, the classification of EEG signals works best when signals in the same “class” (e.g., speed or direction) are similar. In our case, the VEPs evoked by the same speed may relate to various motion directions, and if they also responded to direction, there would be large “within-class variations”.

Second, signal classifications were performed over 11 classes for either speed or direction. This is considered a large number in visual BCI applications where, for the purposes of fidelity, a much smaller set of classes or options (2–4, etc.) are usually preferred [3,41]. This does not mean that we intend to build a BCI system with 11 classes, although it would be more efficient than having fewer classes. Given that chance performance for a two-class task is 50% and for an eleven-class task it is only 9%, it is not surprising that correct direction rates of around 20% are considered “good” performance and the best performance we achieved was 62.5%. Yet a BCI system should be reliable first before we can consider efficiency. We included a large dynamic range for both speed and direction only to get a sense of the sensitivity of EEG signals to the two cues on a large scale.

Previous studies of visual motion typically use a set of gratings that have various spatial frequencies [24]. Our study used a single circle/dot instead, because, under the eye-tracking condition, it would have been difficult to regulate the gaze, e.g., which one and which part of the gratings to be focused on. A single moving circle allowed us to accurately direct the eye movements.

Regarding the choice of EEG signal bandwidth, we choose 0.1–13 Hz because this range included alpha waves in the analysis. It has been shown that alpha waves are important for encoding motion speed [42] as well as spatial locations [4], which match the final locations of the moving object in our study. We tried increasing the frequency range but did not achieve better classification performance (not presented). In the future, the deployment of deep learning algorithms, such as convolutional neural networks (CNNs) [43], may be explored to achieve enhanced results.

### 4.3. Speed and Direction Sensitivity without Eye Movements

When the subjects were required to fix their gaze at the center of the screen while the object was moving, most of them showed higher sensitivity to motion direction than speed. Specifically, seven out of thirteen showed significant performance for speed (Figure 5), whereas ten out of thirteen showed significant performance for direction (Figure 6). When the two cues were compared over all 31 electrodes for each subject, five showed significantly better performance for direction than speed (Figure 8).

It is thus quite puzzling why two subjects (S10 and S11, Figure 5) demonstrated superb performance for speed under the eye-fixed condition. This could not have been caused by accidental eye movements because when the eyes were allowed to track the moving object, no subject, including S10 and S11 themselves, ever demonstrated high performance on a similar scale. Since we were unprepared for this observation during our experimental design, we did not conduct more physiological or psychophysical explorations to study what could have made them more sensitive to motion speed. Future studies may focus on the cause of individual distinctions, such as previously undergoing special athletic training.

### 4.4. Speed and Direction Sensitivity under Eye-Tracking Condition

When our subjects were asked to track the moving object with their gaze, there would presumably have been “EOG contamination” in the recorded EEG signals, especially in the frontal electrodes [5]. When we rescaled direction performance with a larger color scale (Figure 7C), almost all subjects showed the highest performance for the frontal electrodes, with a couple of exceptions. The electrode sites indicate that the higher performance for motion direction under the eye-tracking condition originated from ocular movements. However, when performance from all the electrodes was plotted against the two cues, the majority of subjects showed a negative correlation (Figure 8B), indicating that the two cues were most likely encoded by different electrode sites. In fact, comparing between the brain topography images for speed (Figure 7A) and direction (Figure 7C), we indeed observe a general trend of encoding speed information with more posterior sites, rather than the frontal sites. This finding indicates that EEG signals, rather than EOG, may be more suitable for decoding motion speed.

We should point out that the EEG signals used in all the classifications only span between 0 and 0.5 s after the target movement onset, which covers the duration of the slowest movement (0.5 s). As shown in Figure 3B, there are prominent signal features lasting more than 3 s, presumably caused by the EOG. In fact, including those signal features by extending signal duration from 0.5 s to 1.5 s significantly improved classification performance for speed under the eye-tracking condition (not presented). One reason is that gaze movements generally lag behind the object’s movement with a latency of up to several hundred milliseconds [44]. More importantly, in our experimental protocol, the motion distance was fixed; therefore, the time it took for each movement with a particular speed varied, followed by a 1 s holding period. Had we extended the signal being analyzed to more than 0.5 s, we could have potentially picked up those factors.

Apart from the EOG effect in the eye-tracking condition, there are fundamental differences in retinal motion processing between situations when the eyes are fixed and when they are actively pursuing a moving object. For example, when an object is moving at a slow speed, its image is more or less stationary on the retina [6]. In the present study, the time duration for the object to complete the movement from the center to the periphery of the large circle varied between 0.1 s and 0.5 s. The faster the movement, the more difficult it is for the object’s image to stay at the center of the retina, which may bring additional complexity to the problem.

### 4.5. Conclusions

In conclusion, our study revealed that EEG contains important signal features that correlate to both the angle and speed of a moving visual object. A linear machine learning classifier was used to quantify sensitivity to both cues. When the gaze was fixed at the center of the view, sensitivities to both cues were comparable. When the gaze was tracking the object’s movement, sensitivity to angle was predominantly better than sensitivity to speed. Our finding provided insights on the brain’s responses to motion-related visual cues in a complex presentation paradigm and examined the potentials of those cues in the application of visual BCIs.

It should be noted that EEG analysis, and thus the derived conclusions, may vary with the choices of signal features and processing methods. For example, if only a certain frequency wave, such as the alpha wave, is used in the analysis, different conclusions may be derived. Here, the frequency we used (0–13 Hz) included delta, theta, and alpha waves, because we found that further extending it to higher frequencies did not improve classification results, whereas limiting it to a smaller range would impair the classification results. We also applied the classification algorithm to entire temporal waveforms, because we were unable to derive significant results using frequency analysis (such as computing the power of a certain frequency band). Nevertheless, other researchers may have better strategies for analyzing data. In addition, future studies should explore the two cues with other types of visual stimulus presentations and improved recording methods, such as high-density EEG or near-infrared spectroscopy.

## Data Availability

The data presented in this study are available on request from the corresponding author. The data are not publicly available due to ethical requirements.

## References

[1] Farwell L., Donchin E. (1988). Talking off the top of your head: Toward a mental prosthesis utilizing event-related brain potentials. Electroencephalogr. Clin. Neurophysiol..

[2] Rao R. (2019). Brain-Computer Interfacing: An Introduction.

[3] Mohebbi A., Engelsholm S.K., Puthusserypady S., Kjaer T.W., Thomsen C.E., Sorensen H.B. A brain computer interface for robust wheelchair control application based on pseudorandom code modulated Visual Evoked Potential. Proceedings of the 2015 37th Annual International Conference of the IEEE Engineering in Medicine and Biology Society (EMBC).

[4] Samaha J., Sprague T.C., Postle B.R. (2016). Decoding and reconstructing the focus of spatial attention from the topography of alpha-band oscillations. J. Cogn. Neurosci..

[5] Li X., Guan C., Zhang H., Ang K.K. (2016). Discriminative Ocular Artifact Correction for Feature Learning in EEG Analysis. IEEE Trans. Biomed. Eng..

[6] Freeman T.C.A., Cucu M.O., Smith L. (2018). A preference for visual speed during smooth pursuit eye movement. J. Exp. Psychol. Hum. Percept. Perform..

[7] Wolpaw J.R., Birbaumer N., McFarland D.J., Pfurtscheller G., Vaughan T.M. (2002). Brain–computer interfaces for communication and control. Clin. Neurophysiol..

[8] Nicolas-Alonso L.F., Gomez-Gil J. (2012). Brain Computer Interfaces, a Review. Sensors.

[9] Chai R., Ling S.H., Hunter G.P., Tran Y., Nguyen H.T. (2013). Brain–computer interface classifier for wheelchair commands using neural network with fuzzy particle swarm optimization. IEEE J. Biomed. Health Inform..

[10] Besserve M., Jerbi K., Laurent F., Baillet S., Martinerie J., Garnero L. (2007). Classification methods for ongoing EEG and MEG signals. Biol. Res..

[11] Balakrishnama S., Ganapathiraju A., Picone J. Linear discriminant analysis for signal processing problems. Proceedings of the Proceedings IEEE Southeastcon’99. Technology on the Brink of 2000.

[12] Sosulski J., Tangermann M. (2022). Introducing block-Toeplitz covariance matrices to remaster linear discriminant analysis for event-related potential brain–computer interfaces. J. Neural Eng..

[13] Sosulski J., Kemmer J.-P., Tangermann M. (2020). Improving Covariance Matrices Derived from Tiny Training Datasets for the Classification of Event-Related Potentials with Linear Discriminant Analysis. Neuroinformatics.

[14] Finke M., Billinger M., Büchner A. (2017). Toward Automated Cochlear Implant Fitting Procedures Based on Event-Related Potentials. Ear Hear..

[15] Kau S., Strumpf H., Merkel C., Stoppel C., Heinze H.-J., Hopf J.-M., Schoenfeld M. (2013). Distinct neural correlates of attending speed vs. coherence of motion. NeuroImage.

[16] Rasulo S., Vilhelmsen K., van der Weel F.R., van der Meer A.L.H. (2021). Development of motion speed perception from infancy to early adulthood: A high-density EEG study of simulated forward motion through optic flow. Exp. Brain Res..

[17] Webster J.G. (2009). Medical Instrumentation: Application and Design.

[18] Zhou W., Gotman J. (2009). Automatic removal of eye movement artifacts from the EEG using ICA and the dipole model. Prog. Nat. Sci..

[19] Goldring J.E., Dorris M.C., Corneil B.D., Ballantyne P.A., Munoz D.R. (1996). Combined eye-head gaze shifts to visual and auditory targets in humans. Exp. Brain Res..

[20] Romero M.C., Castro A.F., Bermudez M.A., Perez R., Gonzalez F. (2007). Eye dominance and response latency in area V1 of the monkey. Vis. Neurosci..

[21] Sharma A., Paliwal K.K. (2014). Linear discriminant analysis for the small sample size problem: An overview. Int. J. Mach. Learn. Cybern..

[22] Dong Y., Gai Y. (2021). Speech Perception with Noise Vocoding and Background Noise: An EEG and Behavioral Study. J. Assoc. Res. Otolaryngol..

[23] Lock R.H., Lock P.F., Morgan K.L., Lock E.F., Lock D.F. (2017). Lock, Statistics: Unlocking the Power of Data.

[24] Pasternak T., Tadin D. (2020). Linking Neuronal Direction Selectivity to Perceptual Decisions About Visual Motion. Annu. Rev. Vis. Sci..

[25] Trappenberg T.P. (2022). Fundamentals of Computational Neuroscience.

[26] Hubel D.H., Wiesel T.N. (1962). Receptive fields, binocular interaction and functional architecture in the cat's visual cortex. J. Phys..

[27] Holub R.A., Morton-Gibson M., Ziskind A.J., Emondi A.A., Kurgansky A.V., Rebrik S.P., Miller K.D., Nowak P., Dobbins A.C., Gawne T.J. (1981). Response of Visual Cortical Neurons of the cat to moving sinusoidal gratings: Response-contrast functions and spatiotemporal interactions. J. Neurophysiol..

[28] DeAngelis G.C., Uka T. (2003). Coding of horizontal disparity and velocity by MT neurons in the alert macaque. J. Neurophysiol..

[29] Maunsell J.H.R., Van Essen D.C., Schledde B., Galashan F.O., Przybyla M., Kreiter A.K., Wegener D., Chaplin T.A., Allitt B.J., Hagan M.A. (1983). Functional properties of neurons in middle temporal visual area of the macaque monkey. I. Selectivity for stimulus direction, speed, and orientation. J. Neurophysiol..

[30] Nover H., Anderson C.H., DeAngelis G.C. (2005). A logarithmic, scale-invariant representation of speed in macaque middle temporal area accounts for speed discrimination performance. J. Neurosci..

[31] Perrone J.A., Thiele A. (2001). Speed skills: Measuring the visual speed analyzing properties of primate MT neurons. Nat. Neurosci..

[32] Duijnhouwer J., Noest A.J., Lankheet M.J.M., Berg A.V.v.D., van Wezel R.J.A. (2013). Speed and direction response profiles of neurons in macaque MT and MST show modest constraint line tuning. Front. Behav. Neurosci..

[33] Lingnau A., Ashida H., Wall M.B., Smith A.T. (2009). Speed encoding in human visual cortex revealed by fMRI adaptation. J. Vis..

[34] Tolhurst D.J., Movshon J.A. (1975). Spatial and temporal contrast sensitivity of striate cortical neurones. Nature.

[35] Gaglianese A., Harvey B.M., Vansteensel M.J., Dumoulin S.O., Ramsey N.F., Petridou N. (2016). Separate spatial and temporal frequency tuning to visual motion in human MT+ measured with ECoG. Hum. Brain Mapp..

[36] Movshon J.A. (1975). The velocity tuning of single units in cat striate cortex. J. Physiol..

[37] Lui L.L., Bourne J.A., Rosa M.G.P. (2007). Spatial and temporal frequency selectivity of neurons in the middle temporal visual area of new world monkeys (*Callithrix jacchus*). Eur. J. Neurosci..

[38] Miura K., Inaba N., Aoki Y., Kawano K. (2014). Difference in visual motion representation between cortical areas MT and MST during ocular following responses. J. Neurosci..

[39] Priebe N.J., Cassanello C.R., Lisberger S.G. (2003). The neural representation of speed in macaque area MT/V5. J. Neurosci..

[40] Priebe N.J., Lisberger S.G., Movshon J.A. (2006). Tuning for Spatiotemporal frequency and speed in directionally selective neurons of macaque striate cortex. J. Neurosci..

[41] Trachel R.E., Clerc M., Brochier T.G. (2015). Decoding covert shifts of attention induced by ambiguous visuospatial cues. Front. Hum. Neurosci..

[42] Shevelev I.A. (1998). Functional importance of α-activity in the visual cortex during recognition of images and movement. Neurosci. Behav. Physiol..

[43] Varone G., Boulila W., Driss M., Kumari S., Khan M.K., Gadekallu T.R., Hussain A. (2024). Finger pinching and imagination classification: A fusion of CNN architectures for IoMT-enabled BCI applications. Inf. Fusion.

[44] Boyer T.W., Wang M. (2018). Direct gaze, eye movements, and covert and overt social attention processes. Attention Perception Psychophys..

